# Prevalence of viral pathogens in a sample of hospitalized Egyptian children with acute lower respiratory tract infections: a two-year prospective study

**DOI:** 10.1186/s42269-022-00790-4

**Published:** 2022-04-13

**Authors:** Amira S. El Refay, Manal A. Shehata, Lobna S. Sherif, Hala G. El Nady, Naglaa Kholoussi, Shams Kholoussi, Nevine R. El Baroudy, Mokhtar R. Gomma, Sara H. Mahmoud, Noura M. Abo Shama, Ola Bagato, Ahmed El Taweel, Ahmed kandeil, Mohamed A. Ali

**Affiliations:** 1 Child Health Department, National Research Centre, 33 El-Bohouth Street (Former El Tahrir St.), PO Box 12622, Dokki, Giza, Egypt; 2 Immunogenetics Department, National Research Centre, Dokki, Egypt; 3 Department of Pediatrics, Faculty of Medicine, Cairo University, Giza, Egypt; 4 Center of Scientific Excellence for Influenza Viruses, National Research Centre, Dokki, Giza, Egypt

**Keywords:** Viral pneumonia, Human coronavirus, Influenza, Lower respiratory tract infection, Egyptian children

## Abstract

**Background:**

Viral pneumonias are a major cause of childhood mortality. Proper management needs early and accurate diagnosis. This study objective is to investigate the viral etiologies of pneumonia in children.

**Results:**

This prospective study enrolled 158 and 101 patients in the first and second year, respectively, and their mean age was 4.72 ± 2.89. Nasopharyngeal swabs were collected and subjected to virus diagnosis by reverse transcription polymerase chain reaction (RT-PCR). Viral etiologies of pneumonia were evidenced in 59.5% of the samples in the first year, all of them were affirmative for influenza A, 2 samples were affirmative for Human coronavirus NL63, and one for Human coronavirus HKU1. In the second year, 87% of patients had a viral illness. The most prevalent agents are human metapneumovirus which was detected in 44 patients (43.6%) followed by human rhinovirus in 35 patients (34.7%) and then parainfluenza–3 viruses in 33 patients (32.7%), while 14 patients had a confirmed diagnosis for both Pan coronavirus and Flu-B virus.

**Conclusions:**

Viral infection is prevalent in the childhood period; however, the real magnitude of viral pneumonia in children is underestimated. The reverse transcriptase polymerase chain reaction has to be a vital tool for epidemiological research and is able to clear the gaps in-between clinical pictures and final diagnoses.

## Background

Acute lower respiratory infections (ALRIs) are one of the prevailing childhood diseases worldwide, despite developments in prevention and management protocols (Marangu and Zar 2019). They are responsible for 1–2 million under-five deaths and 12 million hospitalizations globally (McAllister et al. 2019). Unfortunately, the majority of these fatality rates are reported from developing nations, where socioeconomic issues such as malnutrition are aggravating factors (Ji et al. 2013). In Egypt, it was reported that 10% of below 5 years of child mortalities are caused by pneumonias and other acute respiratory infections (Fadl et al. 2020).

Previous research proved that respiratory infections could be due to either bacterial or viral agents or even both, which makes it difficult to identify the clinical features allowing the physicians to distinguish viral from bacterial diseases at the early onset of the illness (Baroudy et al. 2018; Ho et al. 2018).

Influenza, parainfluenza, respiratory syncytial virus (RSV), and adenoviruses continue to be the main causative agents of respiratory infections. Human metapneumoviruses (hMPVs) have also been documented globally as pathogens of significance (Mourya et al. 2019). Recently with the spread of new emerging viruses, substantial changes in disease burden have occurred and associated mortality was noticeably increased. These are mostly due to pneumonias subsequent to extensions of the viral infections to the lower respiratory tract (Çelik et al. 2020).

In the last decade, molecular detection and sequencing have been directed to enhanced pathogen detection in common respiratory infections in addition to detection of pathogens through outbreak scenarios. Increasing awareness for these and other emerging viruses is essential for providing better care for pediatric patients and informing public health officials of new illnesses (Schuster and Williams 2018). Precise diagnoses of viral infections have been demonstrated to diminish the abuse of antibiotics and decrease the period of hospitalization (Shah et al. 2017).

### Aim of the study

The objective of the current study is to explore the common viral pathogens causing ARI among Egyptian children admitted to the pediatric hospital of Cairo University and clarify the associated clinical characteristics to develop a new algorithm for diagnosis and management of pneumonia in children compatible with the true burden of the disease, instead of the empirical traditional treatment.

## Methods

This prospective study was approved by the ethical committee of the National Research Center and granted with the registration number (16–121). This study was a collaborative work between Abo El-Rish children’s hospital, Cairo University, Child health department, and the Center of Scientific Excellence for Influenza Viruses, National Research Centre.

The study aimed to collect 250 samples (confidence level 95%, margin of error 5%) from children with pneumonia admitted to the Abo El-Rish children’s hospital, Cairo University. Within two years, (2017–2019). We recruited 259 children, 158 of them were recruited in the first year where only influenza and Pan coronaviruses were screened. While 101 were enrolled in the second year, the screening was expanded to include more viral agents (hMPV, HRV, parainfluenza, Pan corona, and Flu-B viruses).

Based on the Integrated Management of Childhood Illness (IMCI) program of WHO, we detected clinical pneumonias in the presence of fast breathing, lower chest in-drawing, inability to drink or feed, or cyanosis (Molyneux 2013). Chest X-ray showing an infiltrate was an add-on diagnostic element. Patient admission records were used to determine eligibility criteria for clinical syndromes of pneumonia. Inclusions criteria are age < 12 years presenting with abrupt onset of fever > 38 °C, coughing or sore throat, difficult breathing, and demanding hospitalization according to the Integrated Management of Childhood Illness (IMCI) criteria for pneumonia (Nguyen et al. 2013). Exclusion criteria are children with other causes of pneumonia rather than infection as aspiration pneumonia. The medical team had a biweekly visit to the pediatric unit to enroll the eligible children.

All the participated children were subjected to written informed consent from the legal guardians after explaining the aim and the procedures of the study.

Clinical examination concentrated on the general examination and local examination of the chest. The general examination included vital signs as temperature, heart rate, and respiratory rate to assess the severity. Local examination of the chest to detect diminished air entry, sibilant rhonchi, and /or crepitation.

### The clinical severity of the participated children was assessed as follows

(1) Children aged two months to five years with coughing or difficult breathing and respiratory rates more than 60/min [infants < 2 months], more than 50/min [infants 2‐12 months], or respiratory rates more than 40 /min [children 1–5 years], or (2) diagnosis of severe pneumonia, according to IMCI criteria for children in the same abovementioned age group and clinical manifestations, besides any of the following broad danger signs: Inability of drinking or breast-feeding, vomit everything, seizures, lethargy or unconsciousness, chest in-drawing, or stridor in quiet children and (3) demanding hospitalization.

The chest X-ray was routinely done for all children with a chest infection, and findings were documented in the patient sheet. Nasal swabs were obtained. Collecting swab samples in medium containing 50% glycerol, 50% phosphate-buffered saline, penicillin (2 × 106 U/L), streptomycin (200 mg/L), and amphotericin B (250 mg/L) (antimicrobial preparations from Lonza, Walkersville, MD, USA). Samples were stored on ice till brought to the laboratory (within 24 h). All samples were kept at – 80 °C till used.

#### Laboratory investigations

##### Screening for coronaviruses (Chu et al. 2011)

Nested RT-PCR was used for screening for Coronaviruses from RNA extracted from collected samples. First round RT-PCR has been carried out by forward primer 5-GGKTGGGAYTAYCCKAARTG-3 and reverse primer 5-TGYTGTS-WRCARAAYTCRTG-3, using (QIAGEN one step RT-PCR kit) a 25 µl reaction combination containing 5 µl of 5X reaction buffer, 1 µl dNTPs, 1 µl enzyme mixture, 1.5 µl (10 P-mole) forward primer, 1.5 µl (10 P-mole) reverse primer, 5 µl ddH2O, and 10 µl of sample RNA. The PCR cycler settings for the amplification were 50 °C for 30 min (reverse transcriptions), then 95 °C for 15 min, 45 cycles of 94 °C for 15 s (denaturation), 48 °C for 30 s (annealing) and 72 °C for one minute (extension) then 72 °C for 10 min (final extension). The PCR products were then undergoing another round PCR, utilizing a new group of primers (forward primer 5-GGTTGG-GACTATCCTAAGTGTGA-3, reverse primer 5-CCATCATCAGATAG-AATCATCAT-3) for amplification of final PCR products (440 bp), by means of (Phusion High Fidelity PCR Master Mix Kit, Thermo Scientific) a 25 µl reaction comprising 12.5 µl of 2 X phusion master mixture, 1.5 µl (10 P mole) forward primer, 1.5 µl (10 P-mole) reverse primer, 7.5 µl H2O, and 2 µl of PCR products. The PCR cycler settings were 98 °C for 30 s then 45 cycles (98 °C for ten seconds, 48 °C for 30 s, 72 °C for 30 s), then 72 °C for 10 min.

To determine a characterized virus, purified amplicons have been prepared for sequencing consuming a Big Dye Terminator Kit 3.1 (Applied Biosystems, Foster City, CA) and underwent additional amplification for 26 cycles at 95 °C, 30 secs; 50 °C, 15 secs; and 60 °C, 4 min. The reaction products were subjected to purification by exclusion chromatography in CentriSep columns, (Princeton Separations, Adelphia, NJ). The recovered constituents were sequenced using an ABI PRISM 310 Genetic Analyzer.

##### Screening of MERS CoV using real-time RT-PCR of UpE and ORF1a

The reaction mixture (25 µl) was prepared by adding 5 µl of extracted RNA to 12.5 µl of 2X Verso OneStep RT-PCR Buffer (Thermo, Scientific), 1 µl of 10 µM of each up E-fwd primer 5’-GCAACGCGCGATTCAGTT-3’ and upE-rev primer 5’-GCCTCTACACGGGACCCATA-3’ and 0.5 µl of 10 µM upE-PrbFAM-CTCTTCACATAATCGCCCCGAGCTCG-BHQ1, 0.25 µl Verso enzyme mix, 1.25 µl RT enhancer, and 3.5 µl H2O. Thermal cycling involved 50 °C for 15 min, then by 95 °C for 15 min and then 45 cycles of 95 °C for 15 s, 60 °C for 60 s. To confirm the positive UpE samples, RT-PCR was carried out for orf1a with the identical protocol using EMC-Orf1a-Fwd primer 5’-CCACTACTCCCATTTCGTCAG-3’EMC-Orf1a-Rev primer CAGTATG TGTAGTGCGCATATAAGCA and EMC-Orf1a-Prb: FAM-TTGCAAATTGGCTT GCCCCC ACT- BHQ1 probe.

#### Detection of Parainfluenza 1, 2, 3, hMPV, hRV, hRSV, and influenza B viruses

RT-PCR was carried out using forward and reverse primers listed in Table 1 using (QIAGEN one step RT-PCR kit) a 25 µl reaction combination containing 5 µl of 5 X reactions buffer, 1 µl dNTPs, 1 µl enzyme blend, 1.5 µl (10 P-mole) forward primers, 1.5 µl (10 P-mole) reverse primers, 10 µl ddH2O, and 5 µl of sample RNA. The PCR cycler settings for amplifying were 50 °C for 30 min (reverse transcription) then 95 °C for 15 min, 40 cycles of 94 °C for 15 s (denaturation), 52 °C for 30 s (annealing) and 72 °C for one minute (extension) followed by 72 °C for 10 min (final extension).

**Table 1:** Primers list for detection of the target viruses

Virus name	Primer’s name	Sequence (5' to 3')	base pairs( bp)	References
Parainfluenza 1	PIS1 +	CCG GTA ATT TCT CAT ACC TAT G	317	Bellau-Pujol et al. (2005)
	PIS1-	CCT TGG AGC GGA GTT GTT AAG		
Parainfluenza virus 2	PIP2 +	AACAATCTGCTGCAGCATTT	508	
	PIP2 −	ATGTCAGACAATGGGCAAAT		
Parainfluenza virus 3	Para3.1	CTCGAGGTTGTCAGGATATAG	189	
	Para3.2	CTTTGGGAGTTGAACACAGTT		
Human metapneumovirus	hmpv 1	CCCTTTGTTTCAGGCCAA	416	Donofrio et al. (1992)
	hmpv 2	GCAGCTTCAACAGTAGCTG		
Human rhinovirus	SRHI1	GCATCIGGYARYTTCCACCACCANCC	549	Bellau-Pujol et al. (2005)
	SRHI2	GGGACCAACTACTTTGGGTGTCCGTGT		
Human Respiratory syncytial virus	vrs P1	GGAACAAGTTGTTGAGGTTTATGAATA TGC	139	Sultani et al. (2015)
	vrs P2	TTCTGCTGTCAAGTCTAGTACACTGTAGT		
Influenza A	M226F	TTGTCCAAAATGCCCTAAATG	335	WHO (2002)
	M561R	GTTCCATAGCCTTTGCCGTAGTGC		
Influenza B	Bvf224	ACATACCCTCGGCAAGAGTTTC	284	WHO (2011)
	Bvr507	TGCTGTTTTGTTGTTGTCGTTTT		
	BYf226	ACACCTTCTGCGAAAGCTTCA	388	
	BYr613	CATAGAGGTTCTTCATTTGGGTTT		

### Statistical methods

The statistical analysis of data was done using SPSS statistics (Statistical Package for Social Sciences) software version 22.0, IBM Corp., Chicago, USA, 2013. After coding and tabulation, the data were summarized using the means and standard deviations for quantitative variables, and numbers and percentages for qualitative variables. Comparisons between groups were done via Chi-square. The levels of significance were obtained at P-value < 0.050, otherwise is insignificant.

## Results

The study enrolled 158 and 101 in the first and second year, respectively, to be 259 (181 males and 78 females), their age ranged between 1 and 12 years, and the mean age was 4.72 ± 2.89 years. In the first year, influenza and Pan coronaviruses were screened, and the results revealed that 98 (59.5%) samples were positive for influenza A, two samples were positive for Human coronavirus NL63 and one sample is positive for Human coronavirus HKU1.

In the second year, the screening was expanded to include more viral agents (hMPV, HRV, parainfluenza, Pan corona, and Flu-B viruses), and 87% of patients had viral illness. The children came from 7 different Egyptian governorates (Cairo, Giza, Qalyubiya, Bany swif, Fayoum, Sinai, and El Minia). The majority of patients were from Giza governorate 37.6%, followed by Cairo governorate 20.8%.

Clinical history of studied cases reported history of asthmatic attacks was the most prevalent in 28.1% of cases, while the history of heart disease and liver diseases was found in 1.5% and 0.77%, respectively. On the other hand, 31.8% of studied cases had a history of poultry exposure.

Fever was the most prevalent clinical manifestation in 95.3% of patients, while cough and sore throat were detected in 71.4% and 75.5%, respectively, as shown in Table 2. Twenty-one children (8.1%) were diagnosed with severe pneumonia according to IMCI criteria.

**Table 2:** Descriptive data of the studied cases

Parameter	First year		Second year		Total	
	No	%	No	%	No	%
*Age (years)*						
** < **1	39	26	8	7.9	47	18.7
1–2	30	20	17	16.8	47	18.7
2–5	45	30	30	30.3	75	29.9
> 5	36	24	44	44.4	82	32.7
*Gender*						
Male	116	73.4	65	64.4	181	66.40%
female	42	26.5	36	35.6	78	33.60%
*Clinical symptoms*						
Fever	158	100	89	88.1	247	95.3
Cough	110	69.2	75	74.3	185	71.4
Sore throat	111	70	77	76.2	188	72.5
*History of heart disease*						
Yes	0	0	4	4	4	1.50%
No	158	?	97	96	255	98.50%
*History of Asthma*						
Yes	40	25.30%	68	32.7	73	28.10%
no	118	74.70%	33	67.3	186	71.90%
*Travel status*						
Yes	0	0	18	17.8	0	0
No	158	100	83	82.2	18	6.90%
*Poultry exposure*						
Yes	82	81.2	0	0	82	31.80%
No	19	18.8	100	100	177	68.20%

### The spectrum of respiratory viruses

**Fig. 1:**
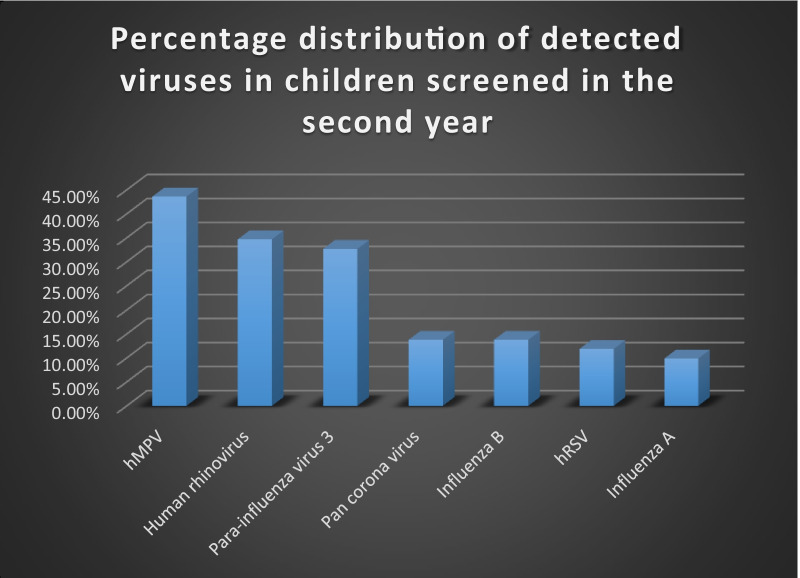
Percentage distribution of viruses detected in children screened in the second year

The most prevalent agents are HMPV which was detected in 44 patients (43.6%) followed by HRV in 35 patients (34.7%) and then parainfluenza–3 viruses in 33 patients (32.7%), while 14 patients were positive for both Pan coronavirus and Flu-B virus (Fig. 1).

The frequency of virologic agents as regards variable age groups detected in the studied children is demonstrated in Table 3. Coinfections with several virologic agents were found in 55 (62.5%) of the study subjects.

**Table 3:** Types of viral infection in different age groups in the second year

	Children < 1 year	Children 1–2 years	Children > 2–5 years	Children > 5 years	Total
hMPV	3(6.8%)	7 (15.9%)	10 (22.7%)	24 (54.5%)	44 (43.6%)
Human rhinovirus	4 (11.4%)	8 (22.9%)	6 (17.1%)	17 (48.6%)	35 (34.7%)
Pan coronavirus	2 (14.3%)	3 (21.4%)	2 (14.3%)	7 (50%)	14(13.8%)
Parainfluenza virus 3	4 (12.1%)	4 (12.1%)	13 (39.4%)	12 (36.4%)	33 (32.7%)
Influenza B	1 (7.1%)	2 (14.3%)	3 (21.4%)	8 (57.1%)	14 (13.8)
hRSV	2 (16.7%)	2 (16.7%)	3 (25%)	5 (41.7%)	12 (11.9%)
Influenza A	0	0	7 (70%)	3 (30%)	10 (9.9%)
Total (age group)	7 (8%)	16 (18.4%)	26 (29.9%)	38 (43.7%)	87 (86.1%)

hMPV: Human metapneumovirus

hRSV: Human respiratory syncytial virus

#### Influence of age on virus detection

The rates of viral infections in children aged less than five 5 years were insignificantly higher than that in older children, and influenza B and PIV 3 were the most common. Viral agents were detected in (43.7%) of subjects who are 5 or more years. Nevertheless, pathogenic viruses were diverse among the age groups. HMPV and hRSV were highly clustered in patients > 5 years (54.5% & 41.7% positive rate), whereas about 40% of the cases with PIV-3 infection were in the age range 2–5 years.

## Discussion

WHO ranks respiratory tract infection as the second leading cause of death around the world for children under the age of 5 years (Bryce et al., 2005). However, vaccines are currently available only for influenza viruses (Fiore et al. 2009). Detection of other specific respiratory viral pathogens provides a useful starting point for ongoing vaccine research.

Pneumonia is a substantial etiology of morbidity and mortality in childhood particularly among children below five years of age (Fadl et al. 2020).

In this study, we explored the viral etiologies of respiratory infections in 259 children, admitted to Abo El-Rish children’s hospital, Cairo University, in a two-year period. Viral etiologies of pneumonia were evidenced in 59.5% of the samples in the first year when screening included only influenza and Pan coronaviruses, while in the second year the rate increased with the increase in the number of viruses covered by the screening to be 87%. Across studies, the percentage distribution of viral etiology of hospitalized cases showed wide variation worldwide varying from 40 to 90% (Bhuyan et al. 2017; Eifan et al. 2017; Caini et al. 2018; Chowdhury et al. 2020). This variation is likely depending upon the number of viruses investigated. In an Egyptian surveillance study (Embarek et al., 2014), about 90% of the enrolled children with severe ARI had a viral etiology, while the prevalence in adults was only 8.8%.

In the current study, the predominant virus was hMPV found in 44 patients (43.6%), and hMPV is a lately recognized paramyxovirus first extracted from hospitalized children with ARI in 2001. HMPV infection occurs at any age, with serious illness manifesting mainly in children under the age of two and the elderly (Falsey et al. 2003). Almost all children by age of five have been infected by hMPV. In a previous Egyptian study, the prevalence rates of hMPV have been estimated from 1.5 to 17.5% (Yahia et al. 2012). HMPV infections commonly cause overt illness; the virus is hardly found in asymptomatic cases. Global detection rates of hMPV infection ranged between 6 and 40% of ARI in children (Shafagati and Williams 2018). These estimates may come in favor of parallel research, and this supports the theory that the hMPV infection is more prevalent in hospitalized children with ALRI.

The rhinovirus was detected in 35 (34.7%), more than 65% of them were cases under 5 years, and it is worth noting that RV presented as a single causative agent in only 4 cases, and this was comparable to another study in Kenya, where RV was one of the most prevalent agents causing severe ARI among children < 5 years; however, the detection rates of it were even higher in their control samples. Moreover, rhinovirus has been the causative agent in an outbreak of serious respiratory infections in infants in Vietnam (Hai et al. 2012) and relation has been evidenced between rhinoviruses and ALRI in earlier literature (Breiman et al. 2015a, b).

RSV is the predominant pathogenic agent in ARI in several research studies, with prevalence estimates ranging from 24 to 64.7% (Shafik et al. 2012; Bin et al. 2019). In the current study, RSV was found in 12 cases (11.9%). This ratio is considered lower than other studies, and this may be owed to that the cases were enrolled from the hospitalized cases.

In the previous studies, the age group usually was 5 years and less as this age usually the age which has the highest hospital admission incidence in community acquired pneumonia (Ostapchuk et al. 2004). We expanded the age group in our study to 12 years, but the mean age in our cases was 4.72 ± 2.89 years which supports the previous data. The epidemiological pattern of respiratory viral illnesses showed that rhinovirus was frequently the most prevalent isolated agent detected in acute respiratory illnesses with prevalence rates between 24 and 50% followed by respiratory syncytial virus (from 22 to 25%) and influenza virus (from 7.2 to 8%) (Feikin et al. 2013; Yu et al. 2015). In a previous study in Egypt done by Fattouh et al. (2011), respiratory syncytial virus was found to be highly predominant (51.9%) in children with combined asthma and ARI.

Coronaviruses infections mostly cause a mild upper respiratory tract infection in human. These viruses have also been detected in diseased mammals and birds. Furthermore, due to their widespread nature, two severe respiratory outbreaks have been associated with coronaviruses. The first was in 2003, Severe Acute Respiratory Syndrome (SARS), and the second one was the Middle-East Respiratory Syndrome (MERS) in 2012 (Raoult et al. 2020). In our present study, two samples were positive for Human coronavirus NL63 and one sample was positive for Human coronavirus HKU1. Coronavirus was detected in other studies. In Egypt, Naga et al. (2020) reported the presence of 3 cases, and in Saudi Arabia, Al-Ayed et al. (2014) found positive samples for NL63 and OC43 in 3.7% of their cases.

It was reported that 20 million patients of ALRI caused by influenza viruses occur annually in children below five years old globally (Simoes et al. 2006). Influenza A virus is well known for its virulence and its association with winter epidemics in temperate zones (Taubenberger 2006).

Influenza pandemics are considered one of the main threats posed by communicable diseases to the human populations (Madhav et al. 2017). In Egypt, human influenza A (H5N1) has been first detected in 2006, with 204 patients diagnosed by the end of 2014 and mortality rates of 35.8% in patients with definite diagnoses (Kayali et al. 2016).

In this study, the influenza virus was detected in about 60% of the samples in the first year, and 10% in the second year, while influenza B was detected in 14%. On the other hand, PIV-3 showed a higher prevalence, as it was detected in 33% of cases, most of them under five years old. This is consistent with data that; PIV is identified as a substantial causative agent of pneumonia in children (Howard et al. 2021). However, a recent Egyptian study reported a lower prevalence rate of PIV-3 (7%). In another Egyptian study, influenza viruses were detected in (12.2%) of preschool children with community acquired pneumonia. (ElSeify et al., 2016).

The contradicting estimates found in several studies could be due to diversity in age group selection, site of recruitment (Inpatient, outpatient, community), geographical areas, laboratory techniques, seasonal variations, criteria of diagnosis, and research period. The introduction of PCR technology endorsed the detection of wide ranges of viruses with more sensitivity and specificity (Shafik et al. 2012).

On the other hand, the clinical inference of positive laboratory findings is sophisticated by the presence of mixed infections as coinfections of respiratory viruses are common (Baroudy et al. 2018).

This study reported high rates of combined viral infections, as it was present in 62.5% of the cases. Combined infection with more than one viral etiology has been reported in previous literature in the Middle East (Bryce et al. 2005; Simoes et al. 2006; Al-Ayed et al. 2014; Eifan et al. 2017), that may complicate the diagnostic methods, as the clinical influence of each virus agent may be obscured (Bryce et al. 2005).

Early detection of the viral etiology of ARIs is very useful for clinicians when treating patients by decreasing unnecessary antibiotics’ prescription, using appropriate antiviral agents, and limiting virus transmission in both outpatients and inpatients (Llor and Bjerrum 2018).

## Conclusions

In conclusion, the results supported the theory that viral infection is prevalent in children; nonetheless, the real magnitude of viral pneumonia in children is underestimated. The management protocol will need to be changed. The reverse transcriptase polymerase chain reaction has to come to be a vital tool for epidemiological research and is able to clear the gaps in-between clinical pictures and final diagnoses.

## Data Availability

The datasets used during the current study are available from the corresponding author on reasonable request.

## Abbreviations

ALRI: Acute lower respiratory tract infection
ARI: Acute respiratory infection
hMPV: Human metapneumovirus
HRV: Human rhinovirus
IMCI: Integrated management of childhood illnesses
PIV: Parainfluenza viruses
RSV: Respiratory syncytial virus
RT-PCR: Reverse transcriptase polymerase chain reaction
WHO: World Health Organization
